# Supplementary material for: A Critical View of the Application of the APEX Software (Aqueous Photochemistry of Environmentally-Occurring Xenobiotics) to Predict Photoreaction Kinetics in Surface Freshwaters
Source: Molecules. 2019 Dec 18;25(1):9. doi: 10.3390/molecules25010009 (PMC7017383; doi:10.3390/molecules25010009)
Supplement: Supplementary file 1 [file molecules-25-00009-s001.zip › 3.2.4_gcc-4.4.0.zip!/3.2.4_gcc-4.4.0/3.2.4_gcc-4.4.0/doc/octave/html/interpreter/Dynamically-Linked-Functions.html]

Dynamically Linked Functions - Untitled


Next: Test and Demo Functions,
Previous: Packages,
Up: Top


---

## Appendix A Dynamically Linked Functions

Octave has the possibility of including compiled code as dynamically
linked extensions and then using these extensions as if they were part
of Octave itself. Octave can call C++ code
through its native oct-file interface or C code through its mex
interface. It can also indirectly call functions written in any other
language through a simple wrapper. The reasons to write code in a
compiled language might be either to link to an existing piece of code
and allow it to be used within Octave, or to allow improved performance
for key pieces of code.

Before going further, you should first determine if you really need to
use dynamically linked functions at all. Before proceeding with writing
any dynamically linked function to improve performance you should
address ask yourself

- Can I get the same functionality using the Octave scripting language only?- Is it thoroughly optimized Octave code? Vectorization of Octave code,
    doesn't just make it concise, it generally significantly improves its
    performance. Above all, if loops must be used, make sure that the
    allocation of space for variables takes place outside the loops using an
    assignment to a matrix of the right size, or zeros.- Does it make as much use as possible of existing built-in library
      routines? These are highly optimized and many do not carry the overhead
      of being interpreted.- Does writing a dynamically linked function represent useful investment
        of your time, relative to staying in Octave?

Also, as oct- and mex-files are dynamically linked to Octave, they
introduce the possibility of Octave crashing due to errors in
the user code. For example a segmentation violation in the user's code
will cause Octave to abort.

- Oct-Files- Mex-Files- Standalone Programs
